# The Treatment and Control of Venereal Diseases

**Published:** 1917-02-03

**Authors:** 


### THE TREATMENT AND CONTROL OF VENEREAL DISEASES.

### From the Standpoint of the Voluntary Hospitals.

ON Friday, January 26, the members of the British Hospitals Association assembled in the old Hall of St. Bartholomew's Hospital to discuss this question, the Right Hon. Viscount Sandhurst, P.C., in the chair. The paper opening the discussion was contributed by the Medical Officer of Health for the City, Dr. J. Howarth, but, owing to the illness of his child, he was unable to be present. In his absence, Dr. Sandilands kindly read the paper.

### DR. HOWARTH'S PAPER.

Dr. Howarth pointed out that where a disease was transmitted directly from man to man, without an intermediate host, adequate precautionary measures were difficult, man being a free agent. In regard to venereal disease, the information necessary to formulate a scheme of prevention was fully available, but the weakness lay in the fact that its success depended on man himself, though it was rendered a little easier by the fact that the method of acquisition of the diseases constituted an offence against the moral code. This, however, caused the victim to wish to hide his shame, hence he did not seek treatment as he should, and so he disseminated the disease among the innocent, the source of infection being hidden until remedies were powerless. The Press, by its frank discussion of the subject, was doing much to inform the public of the dangers wrought by these diseases. In this crusade the voluntary hospitals had recently been called upon for their assistance. The Public Health (Venereal Diseases) Regulations, 1916, set out that every Council must prepare and submit to the Local Government Board a scheme for the treatment at and in hospitals or other institutions of persons suffering from venereal disease. When the Board had approved this scheme the Council must make arrangements for carrying it into effect at the cost of the Council. It also stated that any information obtained under this scheme must be treated as confidential.

As the Royal Commission deprecated the establishment of special institutions for the treatment of these particular diseases—the words "other institutions" being intended to be supplementary to general hospitals—the latter are the bodies to undertake this work which, being a State matter, should be paid for. For the City treatment of these diseases it was proposed to make use of a building not fully used at present, which was originally designed as a hospital; there would be no danger of the special purpose of the patients being known, and City men might be glad of the opportunity of treatment away from their place of residence. It was intended that facilities should be available irrespective of the patient's place of residence, and to give effect to this the Board would repay 75 per cent. of the cost of the scheme to local authorities. Approved hospitals should keep accurate records of attendances of patients from outside districts if the authorities intended to apply for the contribution. In general, the basis of contribution to the hospital would be only the amount which would cover the costs incurred in carrying out the scheme of the authority. For beds, probably the amount paid would be at the rate as ascertained by the annual cost. For work done at the clinics, *per capita* payments would be made; payments based on the number of visits was not likely to be satisfactory. In the City scheme, cases requiring special treatment will have a claim on the bed accommodation of St. Bartholomew's main building, and the clinic would be provided with waiting-rooms, consultation-rooms, dispensary, two wards, two operating or special treatment rooms, and a teaching and demonstration room. All special work would be carried out under the direction of the hospital pathologist. The whole scheme would be under the direct administration of the hospital authorities.

The question of assistance by general practitioners was still under consideration. The Corporation had suggested as payment a maximum sum of £1,800 for the first year, exclusive of the cost of salvarsan or the approved substitutes. The capital expenditure necessary for equipment and alterations—estimated at about £1,000—would be borne by the Corporation, these amounts being subject to the Government grant, if approved by the Board. Arrangements had also been made at St. Bartholomew's Hospital, apart from the City scheme, for putting into practice what might be called a preventive treatment, a subject which Sir Bryan Donkin had recently ventilated in the *Times*. The value of early treatment was very great. Hospitals ought not to embark on this new venture with any limited outlook, and treatment must not be the sole object of interest: hospital authorities should consider all the associated problems, and be prepared to offer opinions based on their acquired experience.

The author would like to see hospitals become the real centres of medical life. The staff also had obligations in this connection, for a wide gulf separated the specialist from the practitioner, and the transfer of the former's special knowledge was not as free as could be wished. The proper interpretation of the requirements of the Local Government Board would bring about much improvement in the relations which he discussed. The attendance of medical practitioners at the venereal clinics should be encouraged. With general practitioners, he thought, would lie the greatest possibilities for promoting the success of the scheme, and towards them the hospitals had an obvious duty. The hospital must stand for all that is best in treatment.

He did not think patients in a higher social scale would come to hospitals for this special treatment. The quack in these matters must not be allowed to flourish, and the hospitals should join with the general practitioner in fighting him. The day had passed when a sharp line could be drawn between the functions of curative and preventive medicine. The two main advantages of the present scheme were: that increased publicity would be given to the existence of these diseases, and to the necessity of cure being effected as soon as possible. This would pave the way for a feeling of solicitous concern for the unfortunate patient, instead of the former squeamish aversion, while early treatment would reduce the number of early foci. The present measures were only part of a future scheme, and before reaching finality many controversial matters would require decision. But no subject, in his opinion, called more strongly for energetic action.

### The Discussion.

Mr. R. H. CAIRD remarked that the trouble was that patients were already decidedly syphilitic when they came to the hospital, and what the Act did not provide for was earlier treatment, at a stage anterior to the breaking-out of the rash.

Mr. FRANK HAZELL (Royal Infirmary, Manchester) said the question of financial suitability went at the root of the voluntary system, and at the present time, he believed, the Charity Organisation Society had the matter under discussion. It had been suggested that syphilitic patients should be welcomed to every department of the hospital, without any inquiry being made, and the effect of that on the remainder of the hospital patients would not be to make matters easier. With regard to the question of prevention, the medical profession in this matter seemed to have reversed their ideas altogether in comparison with the attitude on infectious diseases generally: their one idea was to segregate in order to avoid contagion. Surely there was a danger in throwing hospital doors wide open for the reception of these patients. He proceeded to discuss the C.D. Acts, and said the police could often put their hands on the source of all the difficulty—namely, the brothels—but the present law was so extraordinarily framed that unless the police could prove a case brought into Court up to the hilt they dare not bring it at all. The present requirement seemed to be a complete reversal of the attitude previously adopted by the profession.

Dr. SANDILANDS, in reply, said there had been measures in operation in the Army, and especially in India, for a large number of years, consisting of an antiseptic ointment and a permanganate of potash douche, which could be used immediately after exposure to risk. That scheme had been in existence when he was in India in 1900, and was now being very largely introduced all over France and in other countries abroad, and apparently similar schemes were being arranged in London and elsewhere in England. With regard to the question of compulsory isolation, he thought if they were not going to have compulsory isolation, then it was of the very first importance to have adequate treatment, because adequate treatment would curtail the disease not by weeks but by years. He took it that it was for that reason the Royal Commission laid great stress on treatment. He did not at all agree that any reduction in venereal disease would arise from the treatment, but it would curtail very much the period of infectivity. In addition to that, the getting of a man into proper hands and giving him adequate treatment would also, no doubt, have the effect of enabling him to better understand his condition, and he would thus be less likely than in former years to take the risk of infecting others. The main argument, of course, as to compulsory isolation was that it would lead to a great deal more concealment than at present. There were Medical Officers of Health in London who were very strongly in favour of compulsory notification, and did not consider that any advance would be made until it came about. He included himself among those.

Sir WILLIAM COLLINS, in proposing a vote of thanks to the Lecturer, the Chairman, and the Governors, said: Personally, he hoped that the intervention of the State, even to the tune of 75 per cent., of the cases, would not lead to a stereotyped method of treatment, or to arresting progress in further knowledge of the pathology of the disease. Some people would remember what the hospitals of London owed to Lord Sandhurst, the chairman. In 1892 Lord Sandhurst presided over a Committee of the House of Lords on the Needs and Necessities of the London Hospitals, exposing sundry defects in their management, and certainly showing there were some grave needs in the way of financial assistance.

Mr. FRANK HAZELL seconded, and the vote was carried.

Lord SANDHURST, in returning thanks on behalf of Dr. Sandilands and himself, said it was some five or six and thirty years since he had first become a hospital man, and he thought he would carry that or any other meeting with him when he said that whatever difficulties were put up to voluntary hospitals, or any others, they would do their very utmost to meet them in the most satisfactory way possible, provided it was certain they existed for the ultimate good of the sick and necessitous poor. Of course there was the matter of funds, and that was a difficulty with which they were usually faced. After a long experience he had come to the conclusion that the public always had confidence in a good voluntary hospital, and would see it through, sooner or later. If they did not come to the rescue, then something was wrong. With their permission, he proposed to write to the Medical Officer of the City of London, saying how much they regretted that he—although so worthily represented—had not been able to read his paper, and he also proposed to add an expression of sympathy with Dr. Howarth. (Applause.)

